# Interplay Between Duration of Androgen Deprivation Therapy and External Beam Radiotherapy With or Without a Brachytherapy Boost for Optimal Treatment of High-risk Prostate Cancer: A Patient-Level Data Analysis of 3 Cohorts

**DOI:** 10.1001/jamaoncol.2021.6871

**Published:** 2022-01-20

**Authors:** Amar U. Kishan, Alison Steigler, James W. Denham, Almudena Zapatero, Araceli Guerrero, David Joseph, Xavier Maldonado, Jessica K. Wong, Bradley J. Stish, Robert T. Dess, Avinash Pilar, Chandana Reddy, Trude B. Wedde, Wolfgang A. Lilleby, Ryan Fiano, Gregory S. Merrick, Richard G. Stock, D. Jeffrey Demanes, Brian J. Moran, Phuoc T. Tran, Santiago Martin, Rafael Martinez-Monge, Daniel J. Krauss, Eyad I. Abu-Isa, Thomas M. Pisansky, C. Richard Choo, Daniel Y. Song, Stephen Greco, Curtiland Deville, Todd McNutt, Theodore L. DeWeese, Ashley E. Ross, Jay P. Ciezki, Derya Tilki, R. Jeffrey Karnes, Jeffrey J. Tosoian, Nicholas G. Nickols, Prashant Bhat, David Shabsovich, Jesus E. Juarez, Tommy Jiang, T. Martin Ma, Michael Xiang, Rebecca Philipson, Albert Chang, Patrick A. Kupelian, Matthew B. Rettig, Felix Y. Feng, Alejandro Berlin, Jonathan D. Tward, Brian J. Davis, Robert E. Reiter, Michael L. Steinberg, David Elashoff, Paul C. Boutros, Eric M. Horwitz, Rahul D. Tendulkar, Daniel E. Spratt, Tahmineh Romero

**Affiliations:** 1 Department of Radiation Oncology, University of California, Los Angeles; 2 Department of Urology, University of California, Los Angeles; 3 School of Medicine and Public Health, University of Newcastle, Newcastle, New South Wales, Australia; 4 Hospital Universitario de la Princesa, Madrid, Spain; 5 Hospital Son Espases, Palma de Mallorca, Spain; 6 Sir Charles Gairdner Hospital, Perth, West Australia, Australia; 7 Department of Medicine and Surgery, University of Western Australia, Perth, West Australia, Australia; 8 Hospital Universitari Vall d’Hebron, Barcelona, Spain; 9 Department of Radiation Oncology, Fox Chase Cancer Center, Philadelphia, Pennsylvania; 10 Department of Radiation Oncology, Mayo Clinic, Rochester, Minnesota; 11 Department of Radiation Oncology, University of Michigan, Ann Arbor; 12 Radiation Medicine Program, Princess Margaret Cancer Centre, Toronto, Ontario, Canada; 13 Department of Radiation Oncology, University of Toronto, Toronto, Ontario, Canada; 14 Department of Radiation Oncology, Taussig Cancer Institute, Cleveland Clinic, Cleveland, Ohio; 15 Oslo University Hospital, Oslo, Norway; 16 Schiffler Cancer Center, Wheeling Hospital, Wheeling Jesuit University, Wheeling, West Virginia; 17 Department of Radiation Oncology, Icahn School of Medicine at Mount Sinai, New York, New York; 18 California Endocurietherapy Cancer Center, Oakland; 19 Chicago Prostate Cancer Center, Westmont, Illinois; 20 Department of Radiation Oncology and Molecular Radiation Sciences, Johns Hopkins University School of Medicine, Baltimore, Maryland; 21 Department of Radiation Oncology, Program in Solid Tumors, Clínica Universidad de Navarra, Pamplona, Spain; 22 William Beaumont School of Medicine, Oakland University, Royal Oak, Michigan; 23 Department of Urology, Feinberg School of Medicine, Northwestern University, Chicago, Illinois; 24 Department of Urology, University Hospital Hamburg-Eppendorf, Hamburg, Germany; 25 Martini-Klinik Prostate Cancer Center, University Hospital Hamburg-Eppendorf, Hamburg, Germany; 26 Department of Urology, Mayo Clinic, Rochester, Minnesota; 27 Department of Urology, The James Buchanan Brady Urological Institute, Johns Hopkins University School of Medicine, Baltimore, Maryland; 28 Department of Radiation Oncology, West Los Angeles Veterans Health Administration, Los Angeles, California; 29 David Geffen School of Medicine, University of California, Los Angeles, Los Angeles, California; 30 Division of Medical Oncology, Ronald Reagan UCLA Medical Center, University of California, Los Angeles; 31 Department of Medical Oncology, West Los Angeles Veterans Health Administration, Los Angeles, California; 32 Helen Diller Family Comprehensive Cancer Center, University of California, San Francisco; 33 Department of Radiotherapy Oncology, Huntsman Cancer Institute at the University of Utah, Salt Lake City; 34 Division of General Internal Medicine and Health Services Research, University of California, Los Angeles; 35 Department of Human Genetics, University of California, Los Angeles; 36 Seidman Cancer Center, Case Western Reserve University, Cleveland, Ohio

## Abstract

**Importance:**

Radiotherapy combined with androgen deprivation therapy (ADT) is a standard of care for high-risk prostate cancer. However, the interplay between radiotherapy dose and the required minimum duration of ADT is uncertain.

**Objective:**

To determine the specific ADT duration threshold that provides a distant metastasis-free survival (DMFS) benefit in patients with high-risk prostate cancer receiving external beam radiotherapy (EBRT) or EBRT with a brachytherapy boost (EBRT+BT).

**Design, Settings, and Participants:**

This was a cohort study of 3 cohorts assembled from a multicenter retrospective study (2000-2013); a post hoc analysis of the Randomized Androgen Deprivation and Radiotherapy 03/04 (RADAR; 2003-2007) randomized clinical trial (RCT); and a cross-trial comparison of the RADAR vs the *Deprivación Androgénica y Radio Terapía* (Androgen Deprivation and Radiation Therapy; DART) 01/05 RCT (2005-2010). In all, the study analyzed 1827 patients treated with EBRT and 1108 patients treated with EBRT+BT from the retrospective cohort; 181 treated with EBRT and 203 with EBRT+BT from RADAR; and 91 patients treated with EBRT from DART. The study was conducted from October 15, 2020, to July 1, 2021, and the data analyses, from January 5 to June 15, 2021.

**Exposures:**

High-dose EBRT or EBRT+BT for an ADT duration determined by patient-physician choice (retrospective) or by randomization (RCTs).

**Main Outcomes and Measures:**

The primary outcome was DMFS; secondary outcome was overall survival (OS). Natural cubic spline analysis identified minimum thresholds (months).

**Results:**

This cohort study of 3 studies totaling 3410 men (mean age [SD], 68 [62-74] years; race and ethnicity not collected) with high-risk prostate cancer found a significant interaction between the treatment type (EBRT vs EBRT+BT) and ADT duration (binned to <6, 6 to <18, and ≥18 months). Natural cubic spline analysis identified minimum duration thresholds of 26.3 months (95% CI, 25.4-36.0 months) for EBRT and 12 months (95% CI, 4.9-36.0 months) for EBRT+BT for optimal effect on DMFS. In RADAR, the prolongation of ADT for patients receiving only EBRT was not associated with significant improvements in DMFS (hazard ratio [HR], 1.01; 95% CI, 0.65-1.57); however, for patients receiving EBRT+BT, a longer duration was associated with improved DMFS (DMFS HR, 0.56; 95% CI, 0.36-0.87; *P* = .01). For patients receiving EBRT alone (DART), 28 months of ADT was associated with improved DMFS compared with 18 months (RADAR HR, 0.37; 95% CI, 0.17-0.80; *P* = .01).

**Conclusions and Relevance:**

These cohort study findings suggest that the optimal minimum ADT duration for treatment with high-dose EBRT alone is more than 18 months; and for EBRT+BT, it is 18 months or possibly less. Additional studies are needed to determine more precise minimum durations.

## Key Points

**Question:**

What is the optimal minimum duration of androgen deprivation therapy (ADT) when treating high-risk prostate cancer with high-dose radiotherapy?

**Findings:**

This patient-level cohort study of 3 cohorts found a significant interaction between the type of high-dose radiotherapy (external beam radiotherapy with or without a brachytherapy boost) and optimal duration. Prolonging ADT for 18 to 28 months was associated with a 63% reduction in death or metastasis with external beam radiotherapy; when a brachytherapy boost was added, the nonlinear association between ADT and distant metastasis-free survival was broad and spanned 12 months.

**Meaning:**

The findings of this cohort study suggest that patients receiving external beam radiotherapy alone may benefit from ADT durations of 18 months or more; if a brachytherapy boost is added, a duration of 18 months or possibly less may be optimal.

## Teaser

This cohort study explores associations between the duration of androgen deprivation therapy and distant metastasis-free survival in men undergoing external beam radiotherapy with and without a brachytherapy boost for treatment of high-risk prostate cancer.

## Introduction

High-risk prostate cancer is defined by the National Comprehensive Cancer Network (NCCN) as disease with a serum prostate-specific antigen (PSA) level greater than 20 nanograms per milliliter (ng/mL; to convert to μg/L, multiply by 1), a clinical T category 3 or 4, or a Gleason grade group 4 or 5.^1^ Radiation therapy with long-term androgen deprivation therapy (ADT) is an effective standard of care treatment in this setting. Three randomized clinical trials (RCTs) have shown reduced all-cause mortality when external beam radiotherapy (EBRT) was combined with ADT for a longer duration (28-36 months) vs shorter duration (4-6 months).^2,3,4^ A fourth trial showed reduced prostate cancer-specific mortality with an intermediate ADT duration of 18 months vs a shorter duration (6 months).^5^ A single superiority trial failed to demonstrate the superiority of 36 months of ADT over 18 months of ADT.^6^ Therefore, current NCCN guidelines recommend 18 to 36 months of ADT with EBRT for high-risk disease. For patients receiving EBRT with a brachytherapy boost (EBRT+BT), the NCCN guidelines suggest 12 months may be appropriate, based on the favorable progression-free survival rates from the ASCENDE-RT trial.^7^ To our knowledge, this duration has never been analyzed by an RCT.

Given the adverse effects of ADT,^8^ it is commonly underused in real-world settings, with men receiving considerably shorter durations of ADT than indicated.^9,10^ Because of this general underuse and the subsequent lack of duration data to guide decision-making for use of EBRT+BT, we sought to elucidate specific ADT thresholds associated with improved distant metastasis-free survival (DMFS) among patients with high-risk prostate cancer. We interrogated a multi-institutional database of patients with high-risk prostate cancer treated with EBRT or EBRT+BT for varying ADT durations. To evaluate hypotheses generated by this approach, we then analyzed individual patient data from 2 RCTs that evaluated the prolongation of ADT and included patients with high-risk disease who received high-dose EBRT or EBRT+BT.^2,5^

## Methods

This cohort study was reviewed and approved by the ethics review boards of the participating institutions in accordance with the *Tri-Council Policy Statement: Ethical Conduct for Research Involving Humans* (Canada). Informed consent was waived given the retrospective design and use of deidentified data. The study was conducted from October 15, 2020, to July 1, 2021, and followed the Strengthening the Reporting of Observational Studies in Epidemiology (STROBE) reporting guidelines.

### Study Design and Participants

This study included 3 cohorts: 1 retrospective cohort and 2 RCT cohorts. The retrospective cohort was assembled by querying databases from 16 tertiary referral centers for patients with high-risk prostate cancer as defined by NCCN (clinical T category 3 or 4 determined by physical examination, initial PSA [iPSA] level >20 ng/mL, and Gleason grade group 4 or 5) who received definitive treatment with high-dose EBRT or EBRT+BT in 2000 to 2014. High-dose EBRT was defined as equivalent dose in 2-gray (Gy; to convert to rad, multiply by 100) fractions of 74 Gy or higher, assuming an α to β ratio of 3.0 Gy for prostate cancer.^11,12^ For the present analysis, all patients with NCCN-defined high-risk disease were included (2935 of 3366 patients).

Both of the RCT cohorts comprised individual patient data. The Trans-Tasman Radiation Oncology Group 03/04 Randomized Androgen Deprivation and Radiotherapy trial (RADAR)^5^ was a phase 3 RCT conducted across 23 treatment centers in Australia and New Zealand. The study’s eligible patients had clinically localized prostate cancer, either clinical T2b4 or cT2a disease and Gleason grade group 2 disease or higher. Men were assigned equally in a 2 × 2 factorial design to receive 6 or 18 months of ADT, with or without zoledronic acid. Participating centers selected a radiotherapy dosing regimen ranging from 66 to 74 Gy EBRT or 46 Gy EBRT+BT. For the present analysis, all patients with NCCN-defined high-risk disease who received 74 Gy or EBRT+BT were included (384 of 1051 patients).

The *Deprivación Androgénica y Radio Terapía* (Androgen Deprivation and Radiation Therapy; DART) 01/05 trial by the Clinical Investigation in Radiation Oncology Group was a phase 3 RCT conducted across 10 treatment centers in Spain.^2^ The study’s eligible patients had clinically localized prostate cancer with clinical T1c to T3b disease that would be classified as intermediate or high risk by NCCN, with a PSA level less than 100 ng/mL. Men were randomized to receive 4 or 28 months of ADT. The minimum allowable dosage was 76 Gy in 2-Gy doses, with a median delivered dosage of 78 Gy. For the present analysis, men with NCCN-defined high-risk disease who received 28 months of ADT were included (91 of 352 patients).

### Outcomes

The primary outcome for this study was DMFS, defined as time from radiation therapy completion (retrospective cohort) or time from randomization (RADAR and DART) to development of metastasis (typically detected by imaging) or death of any cause. This outcome was chosen because DMFS has been shown to be a surrogate end point for overall survival (OS).^11^ The secondary outcome was OS.

### Statistical Analysis

Continuous variables were summarized using median and IQR ranges. Wilcoxon rank sum and Kruskal-Wallis tests were performed to assess differences between treatment cohorts. Categorical variables were summarized using counts and percentages, and differences between groups were evaluated using the Fisher exact test. For the retrospective cohort, multivariable Cox proportional hazard models were developed to evaluate associations of receiving ADT for less than 6 months, 6 to less than 18 months, or 18 months or more of ADT with DMFS and OS. These specific durations (<6, 6 to <18, and ≥18 months) were chosen because of the durations investigated by the RADAR trial; alternative binning approaches were evaluated as sensitivity analyses. These models were adjusted for the natural logarithm of iPSA (ln[iPSA]), clinical T category, Gleason grade group, age at treatment, treatment type, and any interaction between ADT duration and treatment. This study’s prespecified analysis plan allowed exploration of associations between ADT duration within EBRT and EBRT+BT subgroups individually if *P* < .10 for interaction between ADT duration and treatment type.

Natural cubic splines were used to evaluate a continuous nonlinear relationship between ADT duration and DMFS in the overall cohort, as well as within EBRT and EBRT+BT cohorts. Splines were adjusted for ln(iPSA), clinical T category, Gleason grade group, age (years) at treatment, and duration (months) of ADT; the spline for the overall cohort was also adjusted for treatment type. The optimal degree of freedom for the given splines was decided by evaluating the Akaike information criteria.^13^ The 95% CIs for optimal ADT durations were calculated based on 10 000 bootstraps maintaining the distribution of EBRT and EBRT+BT treatment in the population.

Multivariable Cox proportional hazard models were also developed to evaluate the association between 18 vs 6 months of ADT duration among patients in the RADAR trial who received high-dose EBRT or EBRT+BT, and to evaluate the associations between these outcomes and 6, 18, and 28 months of ADT in a pooled cohort that included patients receiving high-dose EBRT in the RADAR and DART trials. The covariates included in the models were the same as in the retrospective cohort. For the RADAR and DART trial cohorts, all analyses were performed as intention-to-treat analyses, as patient-level ADT duration compliance data were not available.

For evaluating DMFS and OS in the retrospective cohorts and for the cross-trial comparison, including the RADAR high-dose EBRT cohort and patients in the DART trial who received 28 months of ADT, inverse probability of treatment weighting was used to construct covariate-adjusted Kaplan-Meier curves and report 5- and 10-year rates.^14^ For the inverse probability of treatment weighting, propensity scores were estimated using logistic regression for binary outcomes and multinomial logistic regression for trichotomized outcomes, with ADT duration modeled as the outcome and treatment type (EBRT vs EBRT+BT, if applicable), ln(iPSA), clinical T category, Gleason grade group, and age at treatment as independent covariates. Extreme weights above the 99th percentile or below the 1st percentile were truncated to the upper 99% and lower 1%. To access proportionality hazard assumption, we used diagnostic methods based on weighted residuals and visualization tools.^15^ Unadjusted rates were also determined.

Statistical tests were 2-tailed, and *P* values <.05 were considered statistically significant. Data analyses were performed using SAS, version 9.4 (SAS Institute Inc) and R, version 4.0.3 (The R Foundation for Statistical Computing). The data analyses were conducted from January 5 to June 15, 2021.

## Results

The study population totaled 2935 patients (mean age [SD], 69 [63-74] years; 100% male). Demographic information on race and ethnicity was not collected.

### Retrospective Cohort

#### Adjusted Cox Models of DMFS and OS

**Figure 1.**
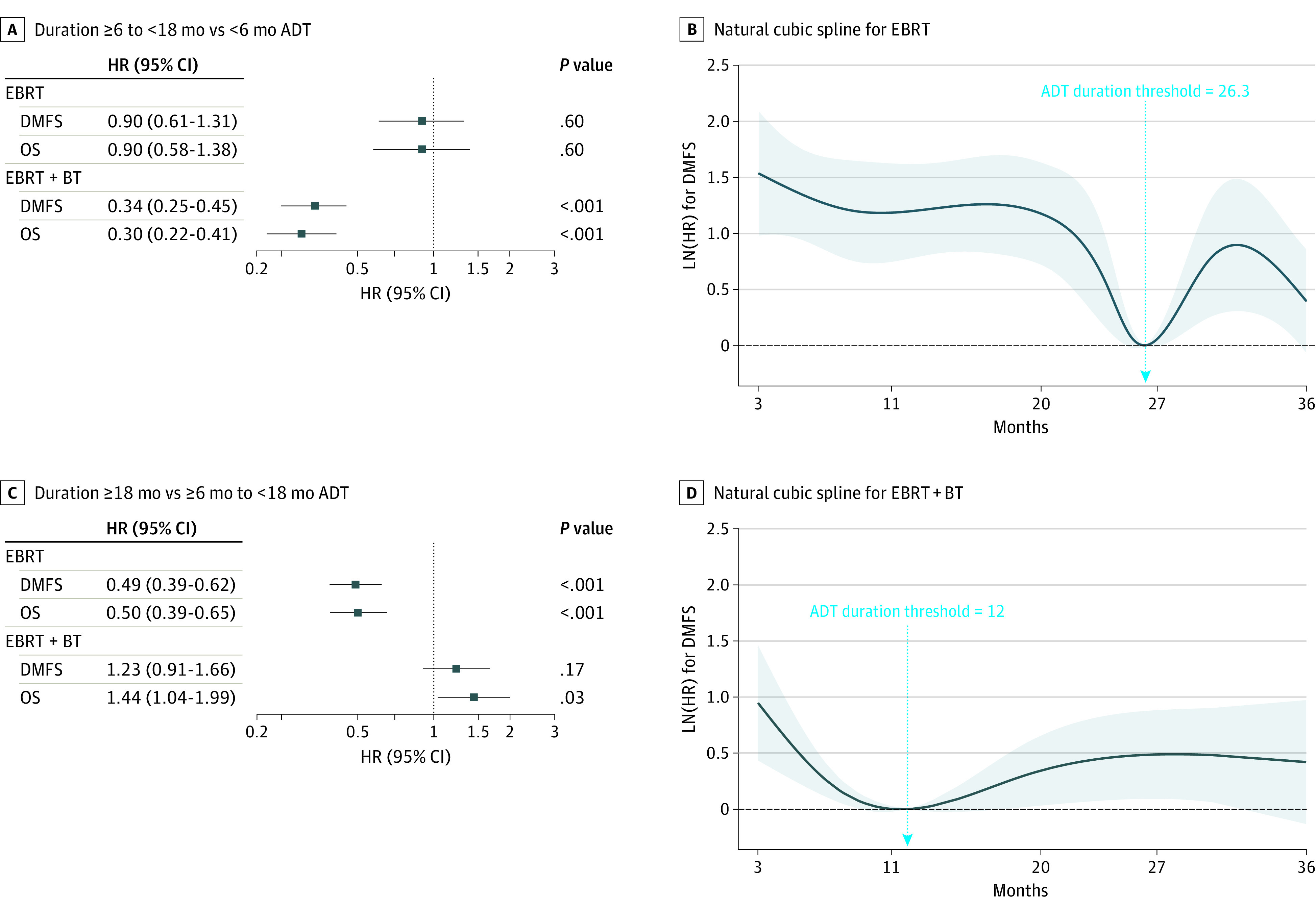
Associations of Androgen Deprivation Therapy Duration With Distant Metastasis-Free Survival and Overall Survival in Men Receiving External Beam Radiotherapy With or Without Brachytherapy Boost A and B, Forest plot comparison ADT duration of 6 to less than 18 months ADT vs less than 6 months. C and D, Comparison of ADT duration of 18 months or more vs 6 to less than 18 months; natural cubic splines evaluating nonlinear associations between ADT duration and DMFS for patients receiving EBRT or EBRT+BT. An arrow on each spline indicates the ADT duration threshold (lowest in [HR]); higher numerical values on the y-axis indicate a less favorable DMFS, and lower values, a more favorable DMFS. Abbreviations: ADT, androgen deprivation therapy; DMFS, distant metastasis-free survival; EBRT, external beam radiotherapy; EBRT+BT, external beam radiotherapy plus brachytherapy boost; HR, hazard ratio; OS, overall survival.

Patient and treatment characteristics for the retrospective cohort are shown in Table 1. The median (range) follow-up period was 6.4 (3.8-9.4) years for patients receiving EBRT and 7.1 (3.7-9.8) years for those receiving EBRT+BT. When trichotomizing the cohort into groups of patients receiving less than 6, 6 to less than 18, and 18 months or more of ADT, significant interactions were found between treatment type and the effect of ADT duration for both DMFS and OS (*P* < .001 for both; eTable 1 in the Supplement). Therefore, associations were explored in subcohorts of patients receiving EBRT or EBRT+BT (Figure 1; eFigures 1 and 2 and eTable 1 in the Supplement). Among patients treated with EBRT, 6 to less than 18 months of ADT was not associated with DMFS (hazard ratio [HR], 0.90; 95% CI, 0.61-1.31; *P* = .58) or OS (HR, 0.90; 95% CI, 0.58-1.38; *P* = .62) compared with less than 6 months of ADT. However, receiving ADT for 18 months or more was associated with improved outcomes compared with ADT for less than 6 months (DMFS HR, 0.44; 95% CI, 0.31-0.63; *P* < .001; OS HR, 0.45; 95% CI, 0.30-0.68; *P* < .001) and 6 to less than 18 months of ADT (DMFS HR, 0.49; 95% CI, 0.39-0.62; *P* < .001; OS HR, 0.50; 95% CI, 0.39-0.65; *P* < .001).

**Table 1.** Patient and Treatment Characteristics in the Retrospective Cohort Data Set

Characteristic	EBRT duration, No. (%)				EBRT+BT duration, No. (%)			
	0	<6 mo	6-<18 mo	≥18 mo	0	<6 mo	6-<18 mo	≥18 mo
No. (%)	478 (26.1)	100 (5.4)	318 (17.4)	931 (51.0)	165 (14.9)	243 (21.9)	297 (26.8)	403 (36.4)
Follow-up, median (IQR), y	6.6 (3.7-10.2)	4.9 (3.2-8.0)	5.6 (3.2-8.2)	6.8 (4.3-9.3)	4.5 (2.3-7.8)	4.6 (2.2-8.5)	7 (4.3-9.3)	8.5 (6.5-11.1)
Age at treatment, median (IQR), y	70 (63.3-75.0)	72.9 (66.3-76.0)	73 (65.9-77.0)	70 (64.0-75.0)	67 (60.0-72.0)	68 (62.5-74.0)	67.2 (61.4-73.4)	67 (62.7-72.0)
cT category								
T1, T2	79 (16.6)	21 (21.4)	56 (17.8)	297 (32.4)	19 (11.5)	68 (28.1)	89 (30.1)	218 (54.2)
T3, T4	398 (83.4)	77 (78.6)	258 (82.2)	621 (67.6)	146 (88.5)	174 (71.9)	207 (69.9)	184 (45.8)
iPSA, median (IQR), ng/mL	14.7 (6.9-27.9)	13 (7.0-22.4)	12.6 (6.8-23.3)	12.3 (6.7-26.4)	9.3 (5.9-20.4)	13.1 (7.7-25.2)	12.1 (6.6-23.4)	23 (11.9-33.4)
iPSA >20 ng/mL								
No	272 (56.9)	63 (63.6)	214 (67.5)	623 (67.1)	117 (70.9)	155 (64.0)	199 (67.0)	160 (39.7)
Yes	206 (43.1)	36 (36.4)	103 (32.5)	305 (32.9)	48 (29.1)	87 (36.0)	98 (33.0)	243 (60.3)
Gleason grade group								
1	41 (8.6)	11 (11 .0)	12 (3.8)	32 (3.4)	25 (15.2)	19 (7.9)	17 (5.8)	54 (13.8)
2	65 (13.7)	12 (12.0)	40 (12.6)	82 (8.8)	19 (11.5)	30 (12.4)	22 (7.5)	39 (9.9)
3	46 (9.7)	10 (10.0)	26 (8.2)	72 (7.7)	11 (6.7)	26 (10.7)	46 (15.6)	106 (27.0)
4	193 (40.5)	43 (43.0)	178 (56.0)	513 (55.1)	85 (51.5)	112 (46.3)	120 (40.7)	111 (28.3)
5	131 (27.5)	24 (24.0)	62 (19.5)	232 (24.9)	25 (15.2)	55 (22.7)	90 (30.5)	82 (20.9)

Abbreviations: EBRT, external beam radiotherapy; EBRT+BT, external beam radiotherapy plus brachytherapy boost; iPSA, initial prostate-specific antigen.

In contrast, among patients receiving EBRT+BT, 6 to less than 18 months of ADT was associated with improved DMFS (HR, 0.34; 95% CI, 0.25-0.45; *P* < .001) and OS (HR, 0.30; 95% CI, 0.22-0.41; *P* < .001) compared with less than 6 months. Receiving ADT for 18 months or more was associated with improved DMFS and OS compared with less than 6 months (DMFS HR, 0.42; 95% CI, 0.32-0.54; *P* < .001; OS HR, 0.43; 95% CI, 0.33-0.56; *P* < .001). However, the analyses did not detect an association between receiving ADT for 18 months or more and improved DMFS compared with 6 to less than 18 months (HR, 1.23; 95% CI, 0.91-1.66; *P* = .17) and the longer duration was associated with inferior OS (HR, 1.44; 95% CI, 1.04-1.99; *P* = .03). When the borders of these trichotomization schemes were adjusted for other permutations (eg, ≤6 vs 6-18 vs >18 months, etc), similar outcomes were seen for patients receiving both EBRT and EBRT+BT (eTable 2 in the Supplement). Because few patients received from 6 to 12 months of ADT (6% in EBRT cohort and 8% in EBRT+BT cohort), we were unable to create more granular categorization schemes that would explore associations for less than 6, 6 to 12, 12 to less than 18, and 18 or more months of ADT. Results of an analysis with a dichotomization around the cut point of 12 months (<12 vs ≥12 months) are shown in eTable 3 in the Supplement and demonstrate that treatment with durations of 12 months or more was associated with improved DMFS and OS in both cohorts.

#### Adjusted Restricted Cubic Splines for DMFS

To quantify the continuous nonlinear relationship between ADT duration and DM, adjusted natural cubic spines were developed. Owing to heightened selection bias related to the use of ADT durations of less than 3 months (eg, for cytoreduction), the splines were fit only on patients receiving 3 months or more of ADT. Splines for the EBRT and EBRT+BT cohorts are shown in Figure 1. For patients receiving EBRT, the optimal ADT duration was 26.3 months (95% CI, 25.4-36.0 months) and for those receiving EBRT+BT, 12 months (95% CI, 4.9-36.0 months).

### Retrospective Cohort Compared With RADAR Cohort

Based on the analyses of the retrospective cohort arms (EBRT and EBRT+BT), we hypothesized that prolonging ADT from 6 to 18 months would improve DMFS among patients receiving EBRT+BT, but not EBRT. To evaluate this, individual patient data from the RADAR trial for patients who received 74 Gy EBRT or EBRT+RT were analyzed to evaluate the association between use of 18 vs 6 months of ADT with DMFS and OS. The median (range) follow-up period was 10 (7.6-11.0) years for EBRT (n = 181 patients) and 11 (9.2-11.9) years for EBRT+BT (n = 203 patients; Table 2). Among all patients, ADT of 18 vs 6 months was not associated with significantly improved DMFS (HR, 0.75; 95% CI, 0.55-1.02; *P* = .07) or OS (HR, 0.75; 95% CI, 0.52-1.06; *P* = .11) overall. There was significant interaction between treatment with EBRT or EBRT+BT and the effect of ADT prolongation on DMFS; thus, subgroup analyses were performed. Among patients receiving EBRT, prolongation of ADT did not significantly improve either DMFS (HR, 1.01; 95% CI, 0.65-1.57; *P* = .90) or OS (HR, 0.88; 95% CI, 0.54-1.45; *P* = .62). Among patients receiving EBRT+BT, prolongation of ADT improved DMFS (HR, 0.56; 95% CI, 0.36-0.85; *P* = .01) but not OS (HR, 0.61; 95% CI, 0.36-1.02; *P* = .06).

**Table 2.** Patient and Treatment Characteristics in the RADAR and DART Cohorts

Characteristic	EBRT duration, No. (%)			EBRT+BT duration, No. (%)	
	RADAR 6 mo	RADAR 18 mo	DART 28 mo	RADAR 6 mo	RADAR 18 mo
No.	85	96	91	101	102
Follow-up, median (IQR)	10.1 (8.2-11.1)	10 (7.3-10.9)	5.4 (4.1-7.1)	11 (8.6-12)	11 (9.6-11.8)
Age at treatment, y	69.5 (63.8-73.7)	69.9 (64.7-74.2)	70.2 (64.4-74.5)	66.6 (60.4-72.3)	66.5 (61.3-71.5)
cT category					
T1, T2	45 (52.9)	53 (55.2)	53 (58.2)	24 (23.8)	27 (26.5)
T3, T4	40 (47.1)	43 (44.8)	38 (41.8)	77 (76.2)	75 (73.5)
iPSA, ng/mL					
Median (IQR)	21.8 (10.8-42.8)	18.5 (10.9-30)	12.4 (7.1-26.2)	16 (10-28)	17 (10-28.6)
>20	46 (54.1)	47 (49)	36 (39.6)	39 (38.6)	41 (40.2)
Gleason grade group^a^					
1	8 (9.4)	7 (7.3)	12 (13.2)	1 (1)	2 (2)
2	21 (24.7)	29 (30.2)	20 (22)	16 (15.8)	12 (11.8)
3	14 (16.5)	12 (12.5)	13 (14.3)	29 (28.7)	22 (21.6)
4	25 (29.4)	23 (24)	33 (36.3)	25 (24.8)	34 (33.3)
5	17 (20)	25 (26)	13 (14.3)	30 (29.7)	32 (31.4)

Abbreviations: DART, *Deprivación Androgénica y Radio Terapía* (Androgen Deprivation and Radiation Therapy) 01/05 trial; EBRT, external beam radiotherapy; EBRT+BT, external beam radiotherapy plus brachytherapy boost; iPSA, initial prostate-specific antigen; RADAR, the Randomized Androgen Deprivation and Radiotherapy 03/04 trial.

^a^ Primary and secondary Gleason grades were not available for the DART cohort.

For the purposes of comparison, we sought to replicate this analysis in the retrospective cohort. Unadjusted and adjusted 5- and 10-year DMFS and OS rates for patients receiving ADT for 4 to 8 months and 16 to 20 months in the retrospective multicenter cohort are presented in Table 3 along with rates for the RADAR cohort. Intervals of ADT duration were chosen in the retrospective cohort to maximize power because ADT was not protocol-specified in this cohort (a distribution of the propensity scores by ADT duration is shown in eFigure 3 in the Supplement). When comparing the DMFS and OS HRs for ADT prolongation in the overall RADAR cohort, as well as in the subgroups receiving EBRT or EBRT+BT, with HRs for a similar prolongation of ADT in the retrospective data set, effect sizes were larger for the EBRT+BT in the RADAR cohort but were otherwise very similar (eFigure 4 in the Supplement). Adjusted Kaplan-Meier curves of DMFS and OS for the retrospective cohort are shown in eFigure 5 in the Supplement.

**Table 3.** Five- and 10-Year Distant Metastasis-Free Survival Rates

ADT duration	% (95% CI) unadjusted				% (95% CI) adjusted^a^			
	5 y		10 y		5 y		10 y	
	EBRT	EBRT+BT	EBRT	EBRT+BT	EBRT	EBRT+BT	EBRT	EBRT+BT
**Distant metastasis-free survival**								
Retrospective data set								
4-8 mo	79.3 (73.4-85.1)	89.4 (85.3-93.4)	49.7 (40.5-58.9)	61.5 (53.6-69.4)	75.4 (68.9-81.9)	89.6 (85.5-93.8)	47.4 (38.2-56.6)	60.9 (53-68.8)
14-20 mo	81.5 (70.9-92.1)	92.9 (88.4-97.4)	50.6 (27.3-73.9)	73.7 (63.4-84.1)	79 (63.8-94.1)	95.4 (88.1-100.0)	37.9 (7.1-68.8)	73.2 (53.9-92.5)
RADAR								
6 mo	78.8 (70.1-87.5)	73.2 (64.5-81.8)	61 (50.6-71.4)	57 (47.2-66.7)	79.6 (70.8-88.3)	72 (63.2-80.8)	61 (50.4-71.6)	55.4 (45.6-65.2)
18 mo	77.8 (69.4-86.2)	84.3 (77.3-91.4)	60.4 (50.5-70.3)	68.6 (59.6-77.6)	76.7 (68.1-85.3)	84.7 (77.6-91.7)	59.6 (49.5-69.6)	69.8 (60.8-78.8)
DART 28 mo^b^	93.5 (87.9-99.1)	NA	NA	NA	93.5 (87.9-99.1)	NA	NA	NA
**Overall survival**								
Retrospective data set								
4-8 mo	84.9 (79.8-90.1)	90.6 (86.8-94.5)	57.2 (47.8-66.6)	66.5 (58.8-74.2)	83.8 (78-89.6)	91 (87.1-94.9)	57.5 (47.9-67)	65.9 (58.1-73.7)
14-20 mo	90.2 (81.9-98.5)	95.3 (91.6-99)	55 (29-81.1)	76.1 (66-86.3)	87.4 (74.8-99.9)	97 (91-100.0)	39.5 (7-72)	75.2 (56.4-94)
RADAR								
6 mo	90.6 (84.4-96.8)	90 (84.2-95.9)	69.3 (59.5-79.2)	72.9 (64.1-81.6)	90.6 (84.2-96.9)	89.1 (83-95.3)	68.5 (58.4-78.5)	71.5 (62.6-80.4)
18 mo	88.4 (81.9-94.8)	95.1 (90.9-99.3)	69 (59.6-78.4)	78.4 (70.4-86.4)	87.3 (80.5-94)	94.8 (90.5-99.2)	68.2 (58.7-77.8)	79.5 (71.6-87.5)
DART 28 mo	96 (91.5-100)	NA	NA	NA	96 (91.5-100)	NA	NA	NA

Abbreviations: ADT, androgen deprivation therapy; DART, *Deprivación Androgénica y Radio Terapía* (Androgen Deprivation and Radiation Therapy) 01/05 trial; EBRT, external beam radiotherapy; EBRT+BT, external beam radiotherapy plus brachytherapy boost; NA, not applicable; PSA, prostate-specific antigen; RADAR, the Randomized Androgen Deprivation and Radiotherapy 03/04 trial.

^a^ Adjustments made using an inverse probability of treatment weighting approach, wherein propensity scores included treatment type (if relevant), ln(initial PSA), clinical T category, Gleason grade group, and age (y) at treatment as independent covariates.

^b^ The median follow-up period for the DART was only 5.3 years; thus, 10-year estimates are not provided.

### RADAR Cohort Compared With DART Cohort 

**Figure 2.**
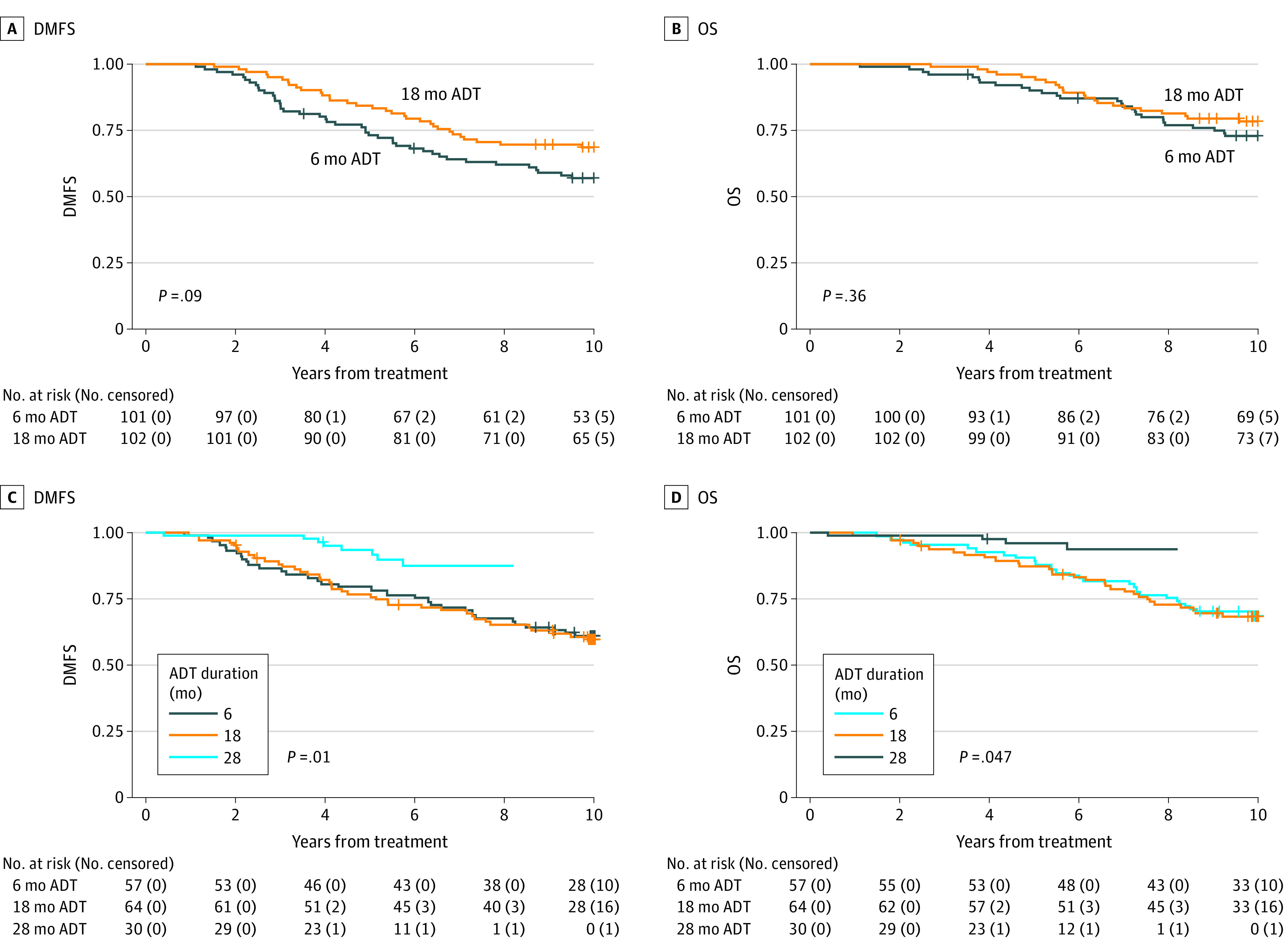
Associations of Longer Duration of Androgen Deprivation Therapy With Distant Metastasis-Free Survival and Overall Survival A and B, Kaplan-Meier curves for DMFS and OS for patients receiving EBRT+BT and 6 or 18 months of ADT on the RADAR trial. Because the RADAR comparison for patients receiving EBRT+BT is the direct randomization from that trial, unadjusted curves are presented. C and D, Adjusted survival curves for DMFS and OS for the cross-trial comparison between patients receiving ADT for 6 and 18 months (RADAR) vs 28 months (DART). Survival curves were adjusted using an inverse probability treatment weighting approach wherein propensity scores included the following independent variables: treatment type (if relevant), ln(iPSA), clinical T category, Gleason grade group, and age (y) at treatment. Abbreviations: ADT, androgen deprivation therapy; DART, *Deprivación Androgénica y Radio Terapía* (Androgen Deprivation and Radiation Therapy) 01/05 trial; DMFS, distant metastasis-free survival; EBRT, external beam radiotherapy; EBRT+BT, external beam radiotherapy plus brachytherapy boost; OS, overall survival; RADAR, the Randomized Androgen Deprivation and Radiotherapy 03/04 trial.

Based on the retrospective data, we further hypothesized that an ADT duration of more than 18 months would improve DMFS and OS in patients receiving high-dose EBRT. Thus, we performed an individual patient data 1-step meta-analysis comparing patients receiving 28 months of ADT in the DART trial with those receiving 6 or 18 months in the RADAR trial. From DART, 91 patients with a median (range) follow-up period of 5.4 (4.1-7.1) years were included; patient characteristics are shown in Table 2. Unadjusted and adjusted survival rates are presented in Table 3, adjusted survival curves are shown in Figure 2, and a forest plot showing associations between 6, 18, and 28 months of ADT and survival outcomes are shown in eFigure 6 in the Supplement. Patients receiving 28 months of ADT had improved DMFS and OS compared with those receiving 6 months (DMFS HR, 0.37; 95% CI, 0.17-0.81; *P* = .01; OS HR, 0.30; 95% CI, 0.10-0.88; *P* = .03). Receiving 28 months of ADT was also associated with improved DMFS compared with 18 months (HR, 0.37; 95% CI, 0.17-0.80; *P* = .01), as was OS (HR, 0.34; 95% CI, 0.12-0.99; *P* = .049). Adjusted 5-year DMFS rates among patients receiving high-dose EBRT were 93.5% (95% CI, 87.9%-99.1%) for 28 months of ADT vs 76.7% (95% CI, 68.1%-85.3%) with 18 months vs 79.6% (95% CI, 70.8%-88.3%) with 6 months. For 5-year OS, adjusted rates were 96% (95% CI, 91.5%-100.0%) with 28 months of ADT vs 87.3% (95% CI, 80.5%-94.0%) with 18 months vs 90.6% (95% CI, 84.2%-96.9%) with 6 months.

## Discussion

To our knowledge, this is the only analysis to interrogate different optimal durations of ADT for patients with high-risk prostate cancer receiving EBRT or EBRT+BT that uses retrospective as well as prospective data from RCTs. The thresholds for ADT duration that were identified by the spline analysis from retrospective data are supported by analysis of individual patient data from the 2 RCTs. If the optimal duration of ADT with high-dose EBRT were to truly be 26.3 months (>18 months), then the effect observed in the RADAR trial (comparing 18 with 6 months) would be small, whereas the effect observed by comparing the 28-month arm of the DART with either the 6- or 18-month arm of RADAR would be significant—this was seen. On the other hand, if the optimal duration of ADT with EBRT+BT were to truly be 12 months, then the effect observed in the RADAR trial would be large, as was the case.

The observation that the optimal duration of ADT for high-risk prostate cancer treated with EBRT might exceed 18 months is contrary to the findings of the Prostate Cancer Study IV trial.^6^ With a median follow-up period of 9.4 years, Nabid and colleagues found that 5-year OS rates were not significantly different for patients receiving ADT for 36 vs 18 months (91% [88%-95%] vs 86% [83%-90%]; *P* = .07) in the context of low-dose EBRT. However, the Prostate Cancer Study IV trial was designed as a superiority trial rather than a noninferiority trial, and only 53% of men assigned to the 36-month arm received that duration of ADT; nearly 25% received less than 21 months. In contrast, 95% of patients in the 28-month arm of the DART study received 28 months of ADT and we knew the exact duration of ADT for patients in the retrospective data set. The findings of the present study raise the possibility that for patients receiving EBRT, 18 months of ADT may be inferior to longer durations.

### Limitations

There are several limitations that must be considered. The multicenter data set is retrospective, and therefore, there are inherent limitations pertaining to selection bias, ecologic bias, and ascertainment bias, as well as to potential differential follow-up, which cannot be mitigated. This is exemplified by the presence of some extreme weights in our propensity score analysis. However, when allowing for some variability in the exact durations of ADT received, the effect sizes of 18 vs 6 months of ADT in the multicenter data set are similar to those seen in the RADAR trial and are actually more conservative with respect to the EBRT+BT cohort. Emerging data suggest that underlying transcriptomic heterogeneity may drive outcomes, and that NCCN-defined high-risk disease itself is a diverse entity.^16,17^ It is possible that the ADT duration effects noted in this study reflect a distribution in biologic aggressiveness that has not been captured in the available data. The RADAR data, while prospective, constitute an exploratory secondary analysis because the choice of treating with EBRT vs EBRT+BT was not randomized. Nonetheless, the focus of this analysis was a comparison within these 2 treatment strata and not between them—and in that regard, the original randomization was preserved. Finally, the comparison between the 28-month arm of the DART trial and the 18-month arm of the RADAR trial is in effect a comparison of 2 parallel prospective cohorts, and thus, does not constitute level I evidence. A strength of this comparison, however, is that the trials in question were contemporaries (RADAR, 2003-2007 and DART, 2005-2010), such that important temporal trends affecting diagnosis, staging, and Gleason grading are less likely to confound the comparison. Nonetheless, the follow-up period was shorter in DART, and it is possible that the difference could erode over time, potentially because of the competing risks. Finally, per-patient ADT duration data were not available for the RCT cohorts. It is possible that a per-protocol analysis might have yielded different results, if compliance to the assigned ADT duration had been poor.

## Conclusions

In conclusion, the findings of this cohort study suggest that the optimal duration of ADT for patients receiving high-dose EBRT may be more than 18 months—implied by the findings in all 3 cohorts. A secondary conclusion, based on the retrospective data set, is that durations of less than 18 months may be sufficient for patients receiving EBRT+BT. Ongoing and future trials will help clarify whether predictive biomarkers can aid in selecting optimal ADT durations; in the interim, individual patient meta-analyses that consider ADT duration data from various relevant trials may be the best available guidance on optimal ADT duration.

## Notes

Supplement. **eFigure 1.** Histograms Depicting Distribution of Use of Various Durations of Androgen Deprivation Therapy in the Multi-Institutional Cohort **eFigure 2.** Associations Between Survival Outcomes and Use of ≥18 months of Androgen Deprivation Therapy vs <6 months of Androgen Deprivation Therapy in the Multi-Institutional Cohort **eTable 1.** Adjusted Cox Models for Distant Metastasis-Free Survival and Overall Survival **eTable 2.** Adjusted Cox Models for Distant Metastasis-Free Survival and Overall Survival Using Alternative Binning Definitions Around 6 and 18 months **eFigure 3.** Distribution of Propensity Scores in the Multi-Institutional Cohort, Stratified by Treatment Type and ADT duration **eTable 3.** Adjusted Cox Models for Distant Metastasis-Free Survival and Overall Survival Using A Dichotomization at 12 Months **eFigure 4.** Associations Between Survival Outcomes and Use of 18 months of Androgen Deprivation Therapy vs 6 months of Androgen Deprivation Therapy in the RADAR Trial with Comparison to Multi-Institutional Cohort **eFigure 5.** Distant Metastasis-Free Survival and Overall Survival Outcomes for Men Receiving 4-6 or 16-20 months of Androgen Deprivation Therapy in the Multi-Institutional Cohort **eFigure 6.** Associations Between Survival Outcomes and Use of 28 months of Androgen Deprivation Therapy in the GICOR Trial vs 6 months or 18 months of Androgen Deprivation Therapy in the RADAR Trial
