# Supplementary material for: Interplay Between Duration of Androgen Deprivation Therapy and External Beam Radiotherapy With or Without a Brachytherapy Boost for Optimal Treatment of High-risk Prostate Cancer: A Patient-Level Data Analysis of 3 Cohorts
Source: JAMA Oncol. 2022 Jan 20;8(3):e216871. doi: 10.1001/jamaoncol.2021.6871 (PMC8778608; doi:10.1001/jamaoncol.2021.6871)
Supplement: Supplement. — eFigure 1. Histograms Depicting Distribution of Use of Various Durations of Androgen Deprivation Therapy in the Multi-Institutional Cohort eFigure 2. Associations Between Survival Outcomes and Use of ≥18 months of Androgen Deprivation Therapy vs <6 months of Androgen Deprivation Therapy in the Multi-Institutional Cohort eTable 1. Adjusted Cox Models for Distant Metastasis-Free Survival and Overall Survival eTable 2. Adjusted Cox Models for Distant Metastasis-Free Survival and Overall Survival Using Alternative Binning Definitions Around 6 and 18 months eFigure 3. Distribution of Propensity Scores in the Multi-Institutional Cohort, Stratified by Treatment Type and ADT duration eTable 3. Adjusted Cox Models for Distant Metastasis-Free Survival and Overall Survival Using A Dichotomization at 12 Months eFigure 4. Associations Between Survival Outcomes and Use of 18 months of Androgen Deprivation Therapy vs 6 months of Androgen Deprivation Therapy in the RADAR Trial with Comparison to Multi-Institutional Cohort eFigure 5. Distant Metastasis-Free Survival and Overall Survival Outcomes for Men Receiving 4-6 or 16-20 months of Androgen Deprivation Therapy in the Multi-Institutional Cohort eFigure 6. Associations Between Survival Outcomes and Use of 28 months of Androgen Deprivation Therapy in the GICOR Trial vs 6 months or 18 months of Androgen Deprivation Therapy in the RADAR Trial [file jamaoncol-e216871-s001.pdf]

Supplemental Online Content

Kishan AU, Steigler A, Denham JW, et al. Interplay between duration of androgen deprivation therapy and external beam radiotherapy with or without a brachytherapy boost for optimal treatment of high-risk prostate cancer: a patient-level data analysis of 3 cohorts. *JAMA Oncol*. Published online January 20, 2022. doi:10.1001/jamaoncol.2021.6871

eFigure 1. Histograms Depicting Distribution of Use of Various Durations of Androgen Deprivation Therapy in the Multi-Institutional Cohort
eFigure 2. Associations Between Survival Outcomes and Use of ≥18 months of Androgen Deprivation Therapy vesus <6 months of Androgen Deprivation Therapy in the Multi-Institutional Cohort
eTable 1. Adjusted Cox Models for Distant Metastasis-Free Survival and Overall Survival
eTable 2. Adjusted Cox Models for Distant Metastasis-Free Survival and Overall Survival Using Alternative Binning Definitions Around 6 and 18 months
eFigure 3. Distribution of Propensity Scores in the Multi-Institutional Cohort, Stratified by Treatment Type and ADT duration
eTable 3. Adjusted Cox Models for Distant Metastasis-Free Survival and Overall Survival Using A Dichotomization at 12 Months
eFigure 4. Associations Between Survival Outcomes and Use of 18 months of Androgen Deprivation Therapy versus 6 months of Androgen Deprivation Therapy in the RADAR Trial with Comparison to Multi-Institutional Cohort
eFigure 5. Distant Metastasis-Free Survival and Overall Survival Outcomes for Men Receiving 4-6 or 16-20 months of Androgen Deprivation Therapy in the Multi-Institutional Cohort
eFigure 6. Associations Between Survival Outcomes and Use of 28 months of Androgen Deprivation Therapy in the GICOR Trial versus 6 months or 18 months of Androgen Deprivation Therapy in the RADAR Trial

This supplemental material has been provided by the authors to give readers additional information about their work.

© 2022 Kishan AU et al. *JAMA Oncol.*

eFigure 1. Histograms Depicting Distribution of Use of Various Durations of Androgen Deprivation Therapy in the Multi-Institutional Cohort

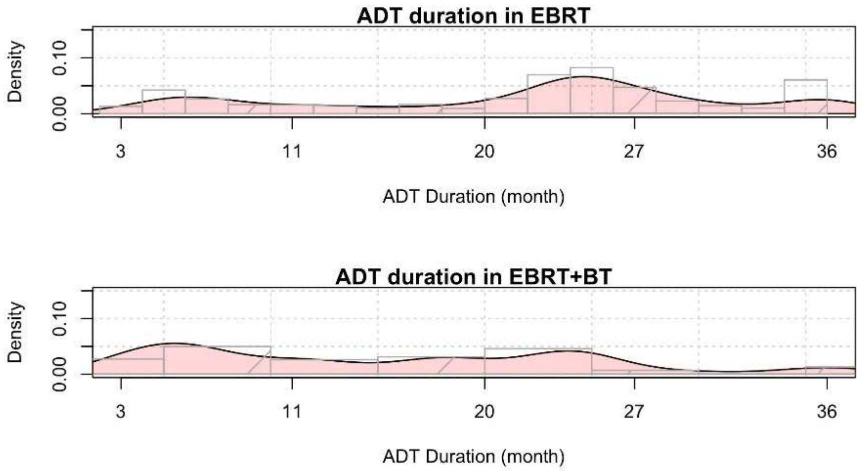

Top row shows density histogram for ADT duration in patients receiving external beam radiotherapy (EBRT), bottom row shows density histogram for ADT duration in patients receiving EBRT+brachytherapy boost (EBRT+BT). ADT, androgen deprivation therapy; EBRT, external beam radiotherapy; EBRT+BT, external beam radiotherapy plus brachytherapy boost.

© 2022 Kishan AU et al. *JAMA Oncol.*

eFigure 2. Associations Between Survival Outcomes and Use of ≥18 months of Androgen Deprivation Therapy versus <6 months of Androgen Deprivation Therapy in the Multi-Institutional Cohort

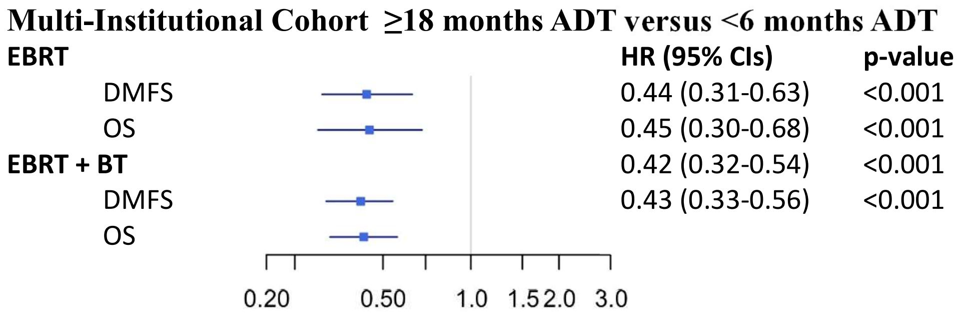

Forest plots showing associations between distant metastasis-free survival (DMFS) and overall survival (OS) and androgen deprivation therapy (ADT) duration in men receiving external beam radiotherapy (EBRT) or EBRT+brachytherapy boost (EBRT+BT). 95% CI; 95% confidence interval; ADT, androgen deprivation therapy; DMFS, distant metastasis-free survival; EBRT, external beam radiotherapy; EBRT+BT, EBRT+brachytherapy boost; HR, hazard ratio.

© 2022 Kishan AU et al. *JAMA Oncol.*

eTable 1. Adjusted Cox Models for Distant Metastasis-Free Survival and Overall Survival

**ADT Duration (months)**	**Distant Metastasis-Free Survival**		**Overall Survival**	
	**HR (95%CI)**	**p-value**	**HR (95%CI)**	**p-value**
**EBRT**				
(≥6 and <18) *vs.* <6	0.9 (0.61 - 1.31)	0.581	0.9 (0.58 - 1.38)	0.615
≥18 *vs.* <6	0.44 (0.31 - 0.63)	<0.001	0.45 (0.3 - 0.68)	<0.001
≥18 *vs.* (≥6 and <18)	0.49 (0.39 - 0.62)	< 0.001	0.5 (0.39 - 0.65)	< 0.001
**EBRT+BT**				
(≥6 and <18) *vs.* <6	0.34 (0.25 - 0.45)	<0.001	0.3 (0.22 - 0.41)	<0.001
≥18 *vs.* <6	0.42 (0.32 - 0.54)	<0.001	0.43 (0.33 - 0.56)	<0.001
≥18 *vs.* (≥6 and <18)	1.23 (0.91 - 1.66)	0.171	1.44 (1.04 - 1.99)	0.026

ADT, androgen deprivation therapy; CI, confidence interval; EBRT, external beam radiotherapy; EBRT+BT, EBRT+brachytherapy boost; HR, hazard radio

© 2022 Kishan AU et al. *JAMA Oncol.*

eTable 2. Adjusted Cox Models for Distant Metastasis-Free Survival and Overall Survival Using Alternative Binning Definitions

ADT Duration (months)	Distant Metastasis-Free Survival		Overall Survival	
	**HR (95%CI)**	**p-value**	**HR (95%CI)**	**p-value**
**Alternative Definition 1:** ≤6 vs. >6 and <18 vs. ≥18				
**EBRT**				
(>6 and <18) *vs.* ≤6	1.23 (0.88 - 1.71)	0.224	1.32 (0.91 - 1.92)	0.144
≥18 *vs.* ≤6	0.54 (0.4 - 0.72)	<0.001	0.58 (0.41 - 0.82)	0.002
≥18 *vs.* (>6 and <18)	0.44 (0.34 - 0.56)	< 0.001	0.44 (0.33 - 0.58)	< 0.001
**EBRT+BT**				
(>6 and <18) *vs.* ≤6	0.43 (0.31 - 0.59)	<0.001	0.4 (0.28 - 0.57)	<0.001
≥18 *vs.* ≤6	0.53 (0.42 - 0.69)	<0.001	0.56 (0.43 - 0.73)	<0.001
≥18 *vs.* (>6 and <18)	1.25 (0.89 - 1.75)	0.199	1.41 (0.98 - 2.03)	0.065
**Alternative Definition 2:** ≤6 vs. >6 and ≤18 vs. >18				
**EBRT**				
(>6 and ≤18) *vs.* ≤6	1.21 (0.87 - 1.67)	0.259	1.3 (0.9 - 1.89)	0.162
>18 *vs.* ≤6	0.53 (0.4 - 0.72)	<0.001	0.58 (0.41 - 0.81)	0.001
>18 *vs.* (>6 and ≤18)	0.44 (0.35 - 0.57)	< 0.001	0.44 (0.34 - 0.58)	< 0.001
**EBRT+BT**				
(>6 and ≤18) *vs.* ≤6	0.4 (0.3 - 0.53)	<0.001	0.4 (0.28 - 0.57)	<0.001
>18 *vs.* ≤6	0.6 (0.46 - 0.78)	<0.001	0.62 (0.47 - 0.81)	<0.001
>18 *vs.* (>6 and ≤18)	1.5 (1.1 - 2.05)	0.011	1.58 (1.13 - 2.19)	0.007
**Alternative Definition 3:** <6 vs. ≥6 and ≤18 vs. >18				
**EBRT**				
(≥6 and ≤18) *vs.* <6	0.89 (0.61 - 1.3)	0.557	0.89 (0.58 - 1.37)	0.599
>18 *vs.* <6	0.44 (0.3 - 0.63))	<0.001	0.45 (0.3 - 0.67)	<0.001
>18 *vs.* (≥6 and ≤18)	0.49 (0.39 - 0.61)	< 0.001	0.5 (0.39 - 0.65)	< 0.001
**EBRT+BT**				
(≥6 and ≤18) *vs.* <6	0.32 (0.25 - 0.42)	<0.001	0.29 (0.22 - 0.39)	<0.001
>18 *vs.* <6	0.47 (0.36 - 0.62)	<0.001	0.47 (0.36 - 0.62)	<0.001
>18 *vs.* (≥6 and ≤18)	1.46 (1.09 - 1.96)	0.01	1.61 (1.18 - 2.18)	0.003

ADT, androgen deprivation therapy; CI, confidence interval; EBRT, external beam radiotherapy; EBRT+BT, EBRT+brachytherapy boost; HR, hazard radio

© 2022 Kishan AU et al. *JAMA Oncol.*

eFigure 3. Distribution of Propensity Scores in the Multi-Institutional Cohort, Stratified by Treatment Type and ADT duration

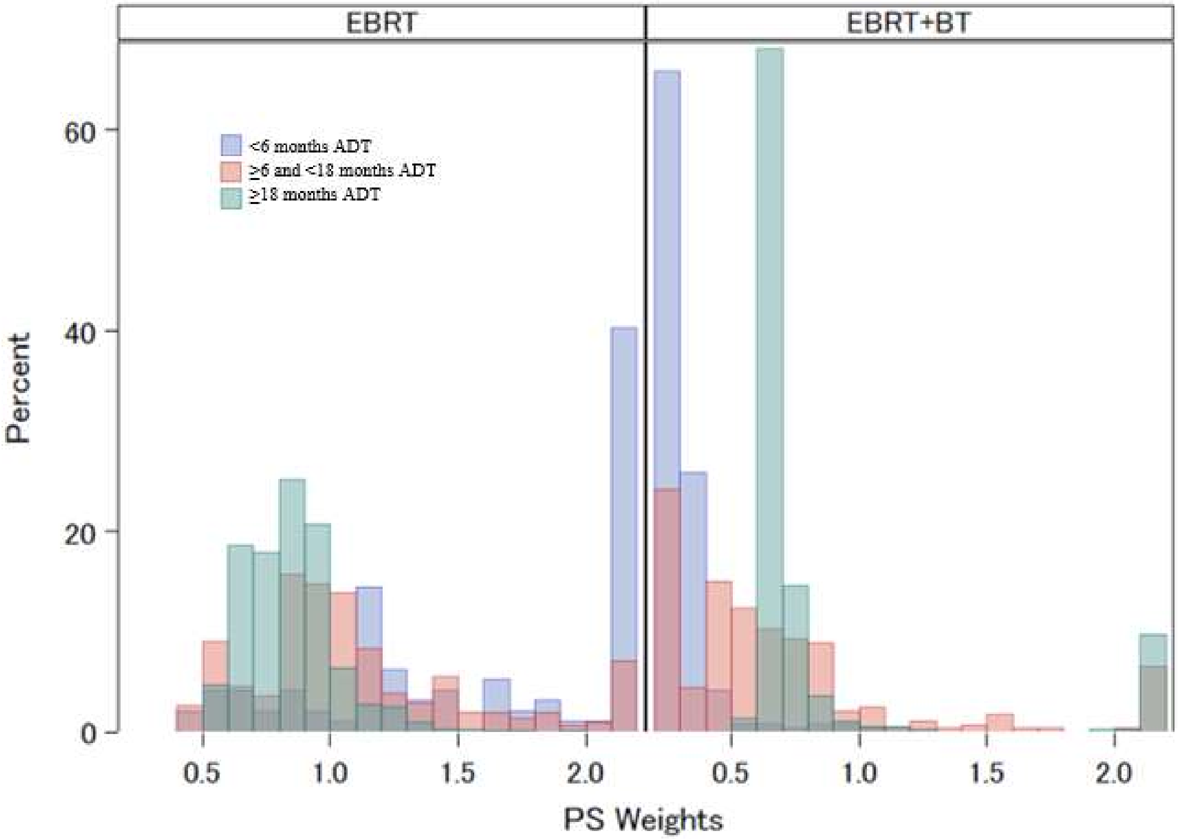

eFigure 3. Distribution of Propensity Scores in the Multi-Institutional Cohort, Stratified by Treatment Type and ADT duration. ADT, androgen deprivation therapy; EBRT, external beam radiotherapy; EBRT+BT, EBRT+brachytherapy boost; PS, propensity score

© 2022 Kishan AU et al. *JAMA Oncol.*

eTable 3. Adjusted Cox Models for Distant Metastasis-Free Survival and Overall Survival Using A Dichotomization at 12 Months

ADT Duration (months)	Distant Metastasis-Free Survival		Overall Survival	
	HR (95%CI)	p-value		HR (95%CI)
**EBRT**				
≥12 *vs.* <12	0.53 (0.42 - 0.66)	<0.001	≥12 *vs.* <12	0.53 (0.42 - 0.66)
**EBRT+BT**				
≥12 *vs.* <12	0.47 (0.36 - 0.6)	<0.001	≥12 *vs.* <12	0.47 (0.36 - 0.6)

ADT, androgen deprivation therapy; CI, confidence interval; EBRT, external beam radiotherapy; EBRT+BT, EBRT+brachytherapy boost; HR, hazard radio

© 2022 Kishan AU et al. *JAMA Oncol.*

eFigure 4. Associations Between Survival Outcomes and Use of 18 months of Androgen Deprivation Therapy versus 6 months of Androgen Deprivation Therapy in the RADAR Trial with Comparison to Multi-Institutional Cohort

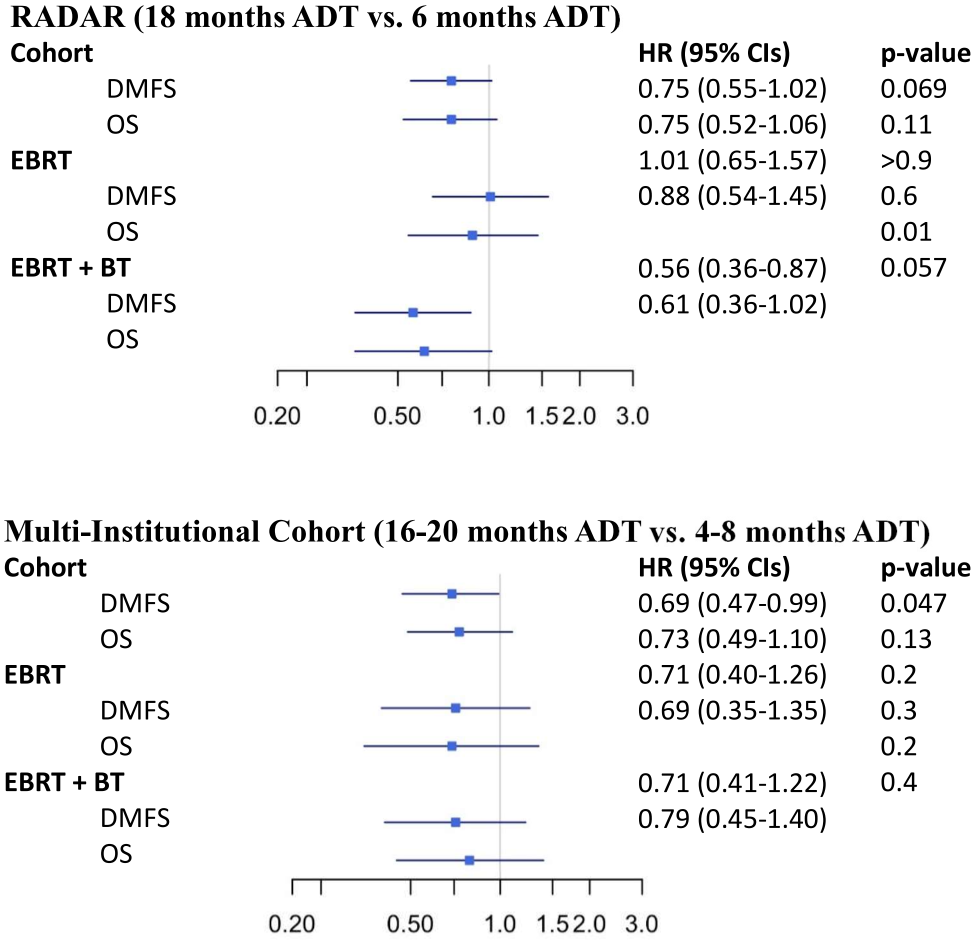

eFigure 4. Associations Between Survival Outcomes and Use of 18 months of Androgen Deprivation Therapy (ADT) versus 6 months of Androgen Deprivation Therapy in the RADAR Trial with Comparison to Multi-Institutional Cohort. Forest plots for patients in the multi-institutional cohort and RADAR. Since ADT duration was not standardized in the multi-institutional cohort, windows of 16-20 months and 4-8 months were chosen to capture patients receiving similar durations to 18 and 6 months, which were protocol-specified for the RADAR trial. 95% CI; 95% confidence interval; ADT, androgen deprivation therapy; DMFS, distant metastasis-free survival; EBRT, external beam radiotherapy; EBRT+BT, EBRT+brachytherapy boost; HR, hazard ratio.

© 2022 Kishan AU et al. *JAMA Oncol.*

eFigure 5. Distant Metastasis-Free Survival and Overall Survival Outcomes for Men Receiving 4-6 or 16-20 months of Androgen Deprivation Therapy in the Multi-Institutional Cohort

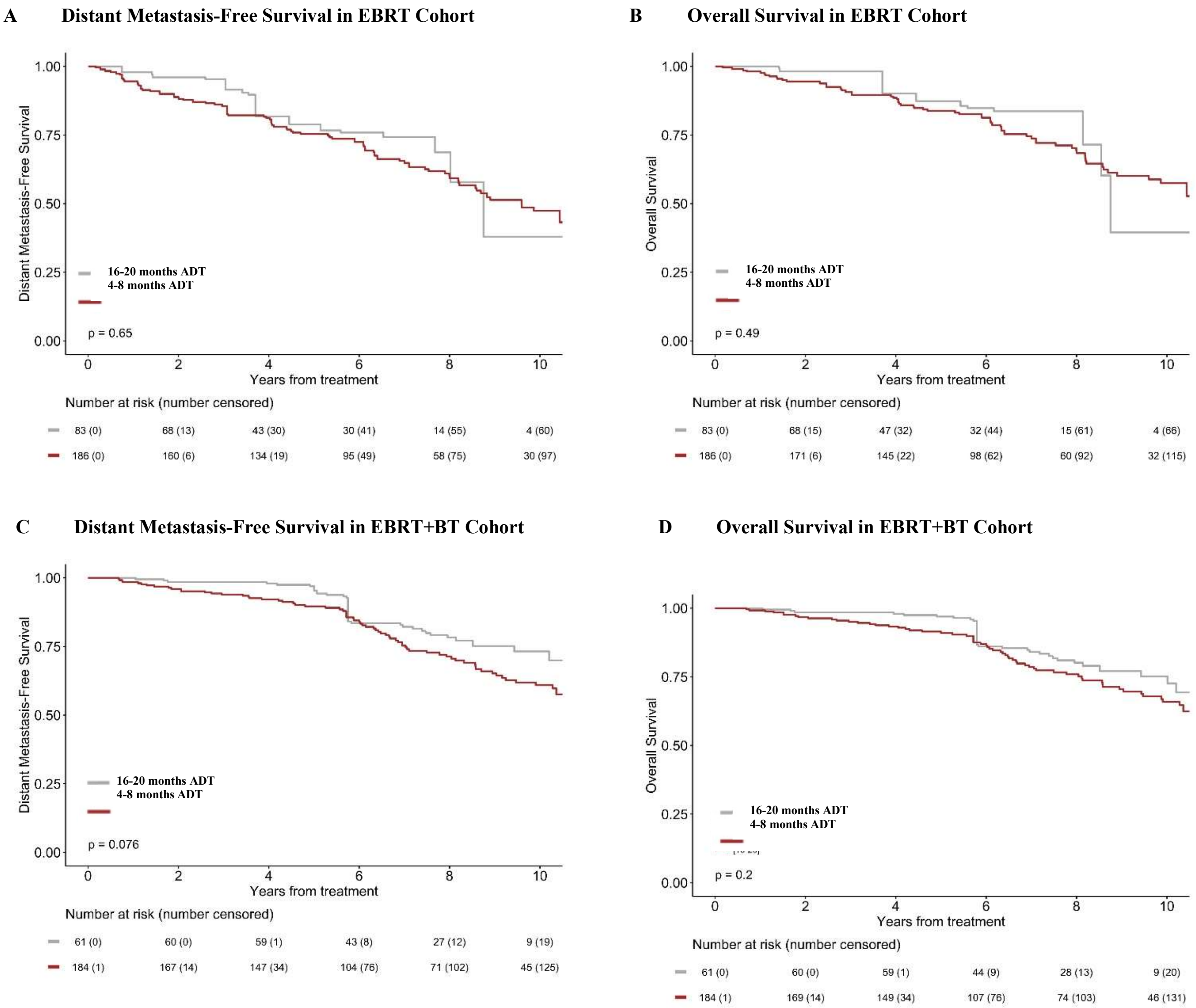

eFigure 5. Distant Metastasis-Free Survival and Overall Survival Outcomes for Men Receiving 4-6 or 16-20 months of Androgen Deprivation Therapy in the Multi-Institutional Cohort. Kaplan-Meier curves for distant metastasis-free survival and overall survival for patients receiving external beam radiotherapy or external beam radiotherapy with a brachytherapy boost. Survival curves were adjusted using an inverse probability treatment weighting approach, wherein propensity scores included treatment type, ln(iPSA), clinical T stage, Gleason grade group, and age at treatment as independent covariates. DMFS, distant metastasis-free survival; EBRT, external beam radiotherapy; EBRT+BT, EBRT+brachytherapy boost; OS, overall survival.

© 2022 Kishan AU et al. *JAMA Oncol.*

eFigure 6. Associations Between Survival Outcomes and Use of 28 months of Androgen Deprivation Therapy in the GICOR Trial versus 6 months or 18 months of Androgen Deprivation Therapy in the RADAR Trial

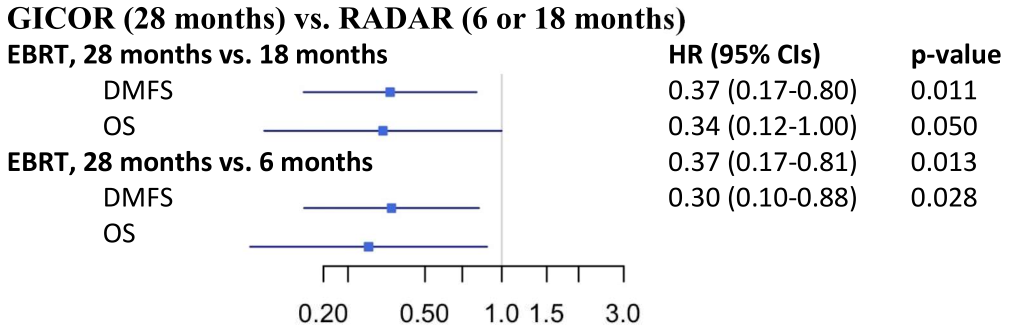

eFigure 6. Associations Between Survival Outcomes and Use of 28 months of Androgen Deprivation Therapy in the GICOR Trial versus 6 months or 18 months of Androgen Deprivation Therapy in the RADAR Trial. Forest plots for a cross-trial comparison between the 28 month arm of GICOR and the 6- and 18- month arms of the RADAR trial. 95% CI; 95% confidence interval; ADT, androgen deprivation therapy; DMFS, distant metastasis-free survival; EBRT, external beam radiotherapy; EBRT+BT, EBRT+brachytherapy boost; HR, hazard ratio.

© 2022 Kishan AU et al. *JAMA Oncol.*
